# The college students' engagement motivations in hot topics on social media: a study based on Q-method from China

**DOI:** 10.3389/fpsyg.2025.1644523

**Published:** 2025-10-17

**Authors:** Ziqi Zhang, Yangsen Huang

**Affiliations:** ^1^School of Marxism, Xuzhou Medical University, Xuzhou, China; ^2^School of Management, Xuzhou Medical University, Xuzhou, China

**Keywords:** engagement in hot topics on social media, motivation of college students, Q methodology, China context, group differences

## Abstract

In the era of mobile internet, college students demonstrate significant enthusiasm for participating in discussions of hot topics on social media platforms. Existing research indicates that this engagement is driven by a variety of internal and external factors, including self-efficacy, psychological empowerment, and the influence of new media technologies. Despite these insights, there remains a scarcity of in-depth studies focusing on the primary motivations behind such behavior, particularly regarding individual differences among students. To address this gap, the present study employs Q methodology—a mixed-method approach combining qualitative and quantitative techniques—to systematically analyze the subjective motivations of college students engaging in online trending topics. Through the analysis of participants' sorting of statement cards and subsequent factor extraction, four distinct engagement profiles were identified: self-referential rationalists, who prioritize practical benefits; values-driven advocates, who are driven by moral and social principles; interest-oriented participants, who seek to share personal opinions; and rights-protection oriented participants, who engage in discourse to protect their pre-existing views or group interests. These findings offer valuable insights for digital citizenship education, student support services, and media literacy programming. Rather than applying uniform approaches, educators and platform designers could develop tailored strategies that acknowledge these varied motivational profiles.

## 1 Introduction

In the era of social media, social hot events or topics are prone to attracting public attention and engagement (Chen et al., 2020). The public who gets rid of the shackles of reality and gains the power of discourse can publish the information that is difficult to distinguish, as well as their views, emotions, and other subjective knowledge, at anytime and anywhere. After the spread of mobile Internet, it will become the network focus of public opinion and social debate at a specific time due to multiple factors such as information convergence, demand superposition, or opinion conflict (Liu, 2023). When the heat of public opinion exceeds the event (topic) itself, the event's resolution or the topic's end may go in two extreme directions.

China has a vast number of internet users, each of whom can receive and send messages on various apps such as WeChat, Weibo, Baidu, TikTok, Bili Bili, Today's Headlines, or online games, and respond through live streaming connections, posts, comments, replies, bullet comments, likes, and other forms (CNNIC, 2024). More and more people are engaging in promoting specific topics to become hot topics on the internet and expanding the scope of their influence (Xu et al., 2021; Xu and Liu, 2018). Of particular concern is that the number of Chinese college students has already reached 46.55 million in 2022, and using mobile phones, pads, and other network terminals for learning, shopping, entertainment, socializing, and receiving information has objectively become the norm in college student life (Ministry of Education of the People's Republic of China, 2023). They are described as “electronic media people” and entering a “networked survival state,” and even some college students have shown a tendency toward “internet addiction” (Shan et al., 2021).

These college students who have yet to mature their values fully and are enthusiastic about engaging in online information are essential participants in the fermentation of online hot events (Zhang and Ding, 2017). They have engaged in various hot topics with their unique perspectives and voices, demonstrating their knowledge level, preferences, needs, attitudes, and values toward the government, to a certain extent promoting the progress of events (topics) and reflecting the progress of human social civilization. However, at the same time, the strong interconnection—weak boundary characteristics of college students' online engagement have also led to negative consequences such as disorderly political participation, group polarization, and significant fluctuations in public opinion, primarily the collective actions that may arise from this, posing a potential threat to social security and stability. The frequent occurrence of large-scale collective behavior extending from the Internet to schools in China is the best example. Therefore, the Chinese government and major universities are paying close attention to the engagement of college students in online hot topics, attempting to explore the different motivations for college students to participate, grasp and intervene in the possible attitudes and directions of college students, and effectively guide college students to engage in an orderly manner, avoiding the occurrence of public opinion events as much as possible.

Scholars have investigated the motivation of college students to engage in hot topics on social media (Zhang and Ding, 2017; Sullivan, 2014). Based on previous research results, individual differences and social environment are the main factors affecting college students' motivation to engage in hot topics on social media (Mu, 2023; Zhang et al., 2021). The results reflected explicitly in self-efficacy, the topic's relevance to them, psychological empowerment, the mediating effect of social support, the impact of new media technology, interests, knowledge, and values. Although existing research mainly focuses on the motivations for college students to engage in hot topics on social media, it has not fully captured the heterogeneity of college student engagement motivations (Sullivan, 2014). That is to say, the uniqueness of each student leads to different combinations of key and less essential motivations when deciding whether to participate and how to engage in hot topics on social media. The Q method requires respondents to rank the decisive roles of all possible motivations to reveal and measure their differentiated engagement motivations. This study used the Q method to analyze the diversity of motivation among Chinese university students engaging in online hot topics, taking Jiangsu Province as an example. The research results indicate that college students mainly engage in online hot topics for four motivations: self-referential rationalists, values-driven advocates, interest-oriented participants, and rights-protection oriented participants. In the next section, we will review the various motivations of college students to engage in hot topics on social media. Then, we described the methods and analysis strategies for data collection. Finally, the results were demonstrated and discussed.

## 2 Motivations that influence college students' engagement in hot topics on social media

Some scholars have discussed and discovered various possible motivations for college students to engage in hot topics on social media, mainly including self-efficacy, the relevance of the topic to “me”, psychological empowerment, the mediating effect of social support, the impact of new media technology, interests, knowledge, and values (Chang et al., 2014; Kim et al., 2014). This section will provide a specific explanation of these six motivations.

### 2.1 Self-efficacy

Scholars have found that young people's self-efficacy affects their willingness to engage in online hot topics (Chang et al., 2014). Self-efficacy is a core concept discussed by Bandura when he proposed the social cognitive theory in 1977. It refers to an individual's confidence in their ability to complete a specific task (Bandura, 1977). Research has found that this feeling and judgment of self-ability, including their level of confidence in their own opinions, their perception of the ability to assume social responsibility, and their perception of the ability to influence the development of social events, serve as key motivations influencing their engagement in hot topics on social media (Krosnick and Petty, 2014; José Alberto Simes Campos and Nofre, 2014). These perceptions are further shaped by social identity processes, wherein individuals derive part of their self-concept from group memberships, influencing their motivations and behavior in collective contexts such as online discourse (Abrams and Hogg, 2010). As some scholars have discovered during surveys, many young people believe that they hope to speak up for the weak and justice and that online engagement is an essential way for them to effectively influence the government and relevant institutions in making fair decisions (Yuste, 2015).

### 2.2 The relevance of the topic to “me”

The discovery of the relevance between online hot topics and oneself is also a possible motivation for young people to engage in hot topics on social media (Kim et al., 2014; Schmitz and Johnson, 2007). The correlation between personal experiences and topics, or the perceived crisis caused by topic content, often drives people to actively engage in online hot topic discussions, share their experiences, or express concerns about similar events. For example, some scholars have found in their research that when individuals have strong concerns about food safety issues, they may be more actively involved in discussions on such topics, supporting, defending, or opposing others on the internet (Xu et al., 2021). In addition, being more sensitive and concerned about topics that involve one's benefit is also an important motivation for people to engage in hot topics on social media. Especially topics closely related to the public, such as public policies and public safety, are more likely to attract public attention and engagement, focusing on expressing attitudes and safeguarding one's benefit. This engagement is not based on fundamental values or beliefs but on people's demands for their benefit (Liu et al., 2016).

### 2.3 Psychological empowerment

Other studies have shown that college students' engagement in online hot topics is related to their psychological empowerment factors (Masur et al., 2014; Yau and Reich, 2019). Various psychological needs drive people to participate, and they hope to explore and build their identities through topic engagement (Arnett, 2023). Especially when the need for self-expression and recognition is not met in the real world, they are more motivated to actively engage in topics in virtual space to showcase themselves, thereby showing their ideal image to others and gaining social recognition (Masur et al., 2014; Chua and Chang, 2016; Forest and Wood, 2012). They will carefully consider the image and its consequences before action, such as being attractive, popular, and thoughtful, to attract others to echo them and gain more social support and recognition (Yau and Reich, 2019; Zhao et al., 2008). Studies indicate that some people engage in online topics only to express and prove their sense of social responsibility (Chae and Park, 2018).

### 2.4 The mediating effect of social support

The mediating role of social support also influences college students' engagement in online hot topics. Social responses triggered around a specific topic can stimulate college students' engagement in action (McCartney et al., 2016). For example, when they are emotionally influenced and mobilized by a group, they may unconsciously participate in and spread the topic. Of course, the mobilization power of opinion leaders is significant (McCartney et al., 2016). Scholars have also analyzed from the perspective of the spiral theory of silence, pointing out that people may measure the atmosphere of public opinion to decide whether to participate and express their opinions (Noelle-Neumann, 1991). Investigation found that nearly half of young people report being pressured by external factors during online engagement, leading to herd behavior (Sun and He, 2014). Compared to the influence of unfamiliar netizens, significant others, such as family and friends, maybe more significant because they need to maintain and develop interpersonal relationships, such as participating in unified actions and discussing the same topic to develop and maintain friendships (Moy et al., 2001; Glynn and Park, 1997; Manago and Melton, 2020).

On the contrary, the corrective action hypothesis may also occur. People with strong opinions on a topic inconsistent with the overall opinion atmosphere will publicly express their opinions to correct the public's conflicting views (Davison, 1983). As emphasizes, such engagement is often motivated by a desire to correct perceived inaccuracies in public opinion, viewing one's own expression as a corrective measure (Rojas, 2010). This viewpoint has been confirmed through surveys and experiments, and some college students may express their opinions loudly in order to correct the opinions of others (Megan et al., 2020). They hope to correct public opinion by engaging in speaking out (Rojas, 2010).

### 2.5 The impact of new media technology

The active application of new media technology by college students is also one of their essential motivations for engaging in hot topics on social media (Delgado et al., 2016). Young people are very interested in new and trendy social environments, so they may be motivated to engage in the topic (Delgado et al., 2016; Cortés-Ramos et al., 2021). This inclination can be further understood through the lens of the Uses and Gratifications (U&G) model, which posits that individuals actively select and use media to fulfill specific psychological and social needs (Wei et al., 2024). Moreover, the obscurity of the internet often weakens the sense of discourse responsibility among college students, which can easily trigger their “chivalrous” mentality and make them more proactive in online engagement. However, this concealment can quickly breed irrational behavior among netizens and may even lead to phenomena such as online violence. In addition, the influence of the “information cocoon” also plays a significant role (Cortés-Ramos et al., 2021). Big data technology profiles the preferences of young people and continuously provides them with homogeneous information, trapping them in an information cocoon and guiding them to engage in a certain topic (Shen, 2020). Such personalized information environments not Narrow but also reinforce existing beliefs, potentially facilitating the spread of mis- or disinformation as users are gratified by content that aligns with their views—a dynamic explained by the U&G framework (Wei et al., 2024). In addition, the power of “hot search” should not be underestimated (Lv, 2022). The ranking of hot search affects and even influences college students' goals and action choices in topic engagement from a technical perspective (Zhou and Sun, 2019). These algorithmic cues create a salient environment that signals social trends and public agenda, thereby triggering participation based on social validation and trend-driven gratification (Wei et al., 2024).

### 2.6 Interests, knowledge, and values

The inherent interests, knowledge, and values of college students are also related to whether they participate in hot topics on social media (Sun and He, 2014; Zhang and Lv, 2019). Among them, interest is the psychological tendency of people to have positive emotional attitudes toward certain things, which makes people prioritize specific affairs or topics and show enthusiasm in action. Its impact on individual engagement behavior is immeasurable (Sun and He, 2014). Hot events can be divided into three categories: political public events, social public events, and non-public events. People are more likely to gather together to pay attention to online political events among these categories of events (Liu et al., 2016). Evidence suggests that young people have a wide range of areas of interest, which affects their motivation to engage in topic discussions (Zhang and Lv, 2019). Of course, the level of understanding of the topic, the level of professional knowledge reserves related to the topic, and the ability to further enrich oneself through engagement are also essential motivations for college students to engage in hot topics on social media (Sun and He, 2014). Last but not least, the inherent values of college students also influence their willingness to engage. According to the value-belief-norm theory, personal values shape beliefs about consequences and feelings of moral obligation, which in turn drive pro-social behaviors such as participating in discussions aligned with those values (Zhang et al., 2025). Their opinions are often driven by the mainstream values of maintaining social fairness and justice (Gao, 2016).

## 3 Method, data, and analysis

This article adopts the Q method to explore and distinguish the dominant motivation of college students to engaging in hot topics on social media. This method is a factor analysis method based on behavioral individuals, advocating for shifting researchers' attention from variables to behavioral individuals (Watts and Stenner, 2012). After arranging and combining various possible motivations among different respondents, it is possible to discover the clustering of motivations for college students to engage in hot topics on social media. This study strictly followed the steps of the Q method program.

In the first stage, build the Q set. We have selected a representative set of statements related to the research topic. These statement sets were derived initially from relevant literature and a large amount of online information. By using induction and deduction methods, 47 statements were compiled. Then, 12 college students organized group discussions to gain more diverse insights into their motivation to engage in hot topics on social media. Through group discussions, we ultimately obtained 61 different statements describing the motivation of college students'engagement. After several rounds of negotiation and discussion among the author, two experts in the relevant field, and student representatives, we finally established a Q set consisting of 32 statements. It should be noted that according to the results of the literature analysis, these 32 statements are further divided into six dimensions and different aspects under each dimension. We have repeatedly tested these statements among college students to ensure that dimension allocation is scientific and semantics are clear.

**Figure 1:**
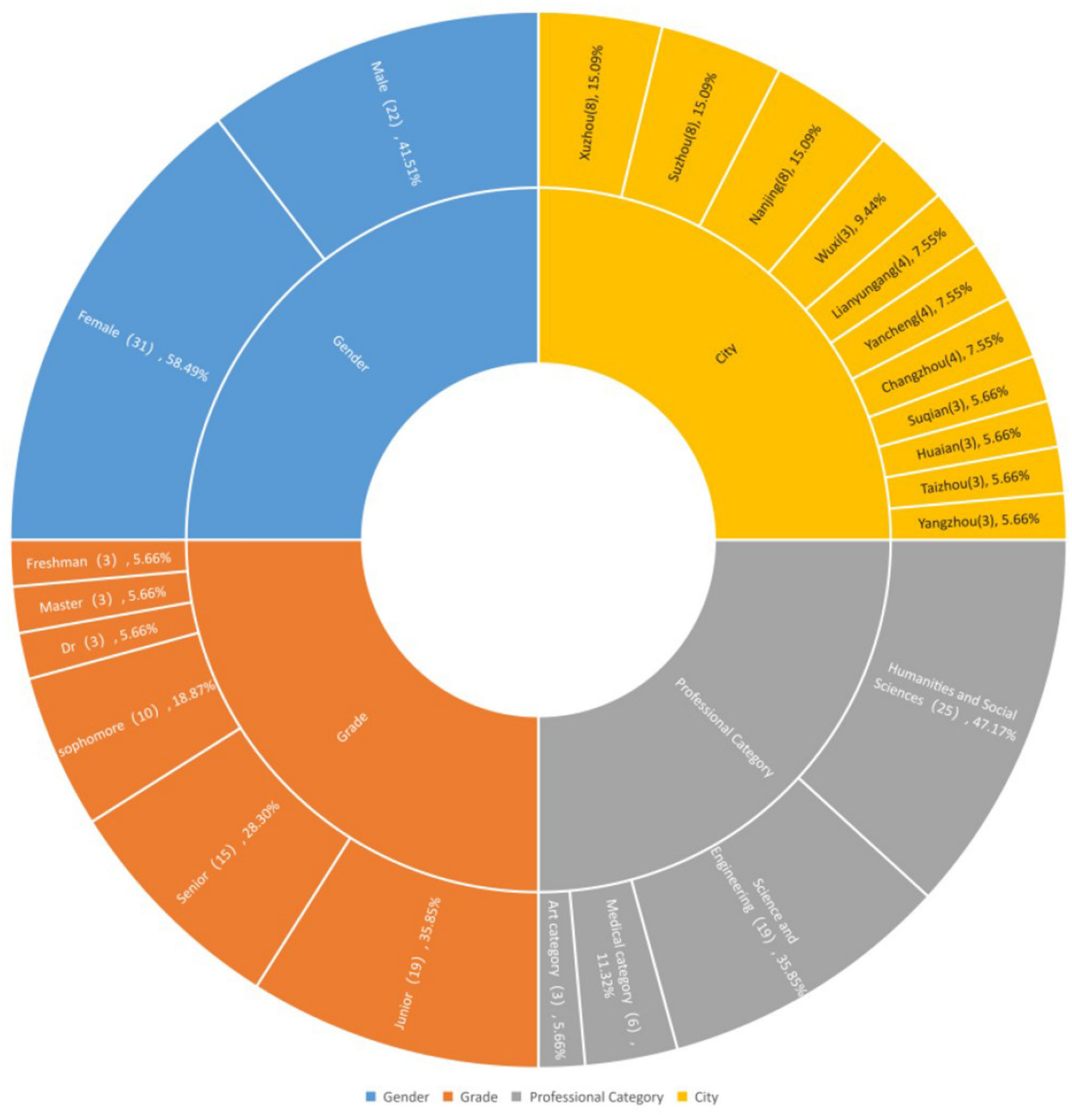
Participants' information (counts per subgroup and percentage %).

In the second stage, the *P*-sample (participant sample) was determined. To ensure the representativeness and generalizability of the findings, this study selected Jiangsu Province as the primary research site. Jiangsu is a pivotal hub for higher education in China, hosting nearly 170 universities of various types (e.g., research-intensive, applied technical, and comprehensive). Its institutions attract a highly diverse student population from across the nation, making it a microcosm of the broader Chinese college student demographic. To capture internal diversity, a multi-stage sampling approach was adopted. We selected universities from 11 cities, including Nanjing, Suzhou, Wuxi, Changzhou, Yangzhou, Taizhou, Xuzhou, Huai'an, Yancheng, Suqian, and Lianyungang. This selection ensured coverage across different regions (southern, central, and northern Jiangsu) and university tiers, enhancing the sample's heterogeneity. Potential participants were initially contacted through university networks. Moreover, college students willing to participate in the research were invited to conduct face-to-face or WeChat video interviews. A snowball sampling approach was also employed, whereby initial participants helped refer others, in order to further increase the diversity of the sample (see Figure 1). Adhering to the small-sample research principles of the Q methodology, which posit that a participant group of 40–60 individuals is sufficient for practical study (Smith et al., 1995), data collection continued until a robust sample within this range was achieved. Ultimately, 53 college students participated in this study. While the author's established connections within the province facilitated logistical access, the primary rationale for selecting Jiangsu was its exemplar status in higher education, which supports the extrapolation of the study's conclusions to similar contexts nationwide.

**Figure 2:**
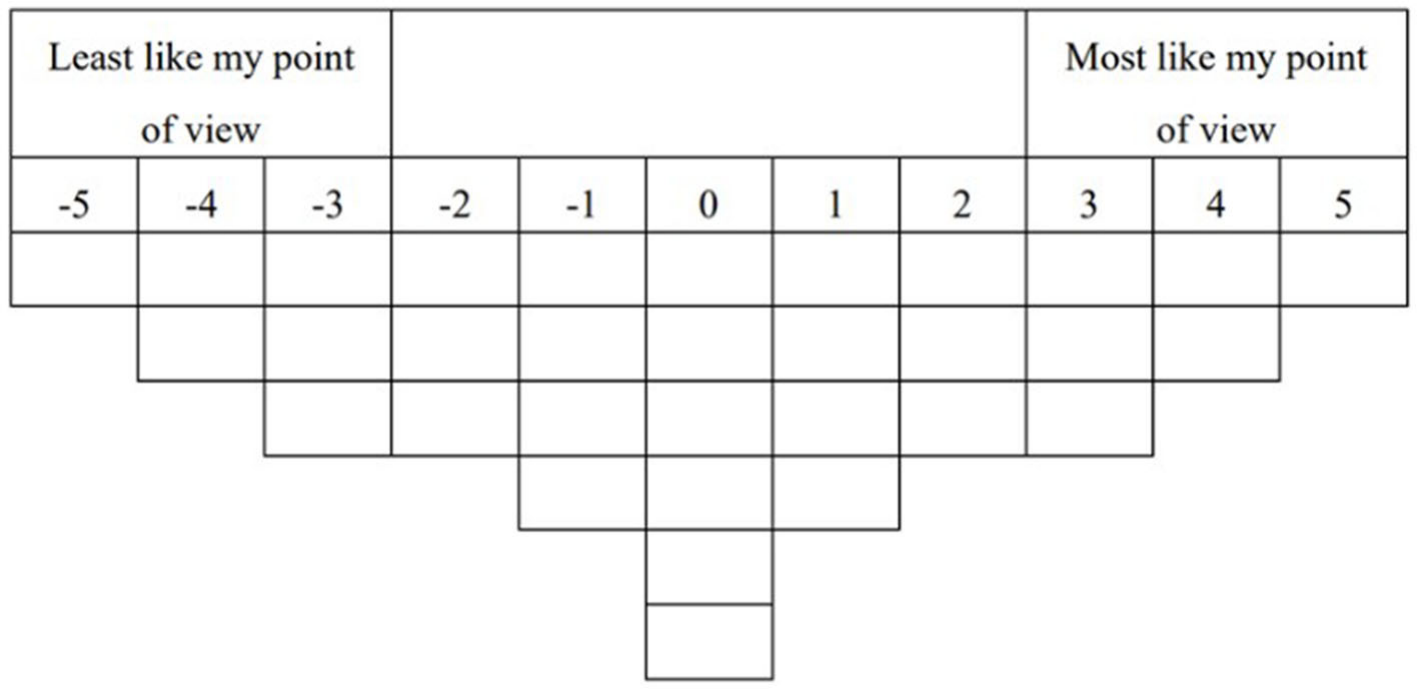
Q sort.

The third stage of the research involved the Q sorting process, which served as the core mechanism for capturing participants' subjective perspectives. Each participant was presented with a set of 32 carefully curated statements (Q-sets) reflecting various attitudes or behaviors related to engaging with hot topics on social media. They were instructed to rank these statements based on their own past experiences or, if lacking direct experience, by imagining plausible scenarios of participation. The sorting was performed according to a predefined quasi-normal distribution grid (see Figure 2), ranging from +5 (most like my point of view) to −5 (least like my point of view). Participants began by reading all statements thoroughly and then initially grouping them into broad categories: those they strongly agreed with, those they strongly disagreed with, and those they felt neutral or uncertain about. They were then required to refine this preliminary arrangement by placing each statement into a specific slot on the sorting board, adhering to the forced distribution pattern. This layout compelled participants to make deliberate comparative judgments, continuously adjusting the order of statements as they reflected on their nuanced opinions. The physical act of moving Q-set cards or digitally arranging items, combined with audible “think-aloud” commentary, provided rich qualitative insight into their decision-making process. Throughout the approximately 30-min session, participants were encouraged to verbalize their reasoning—why certain statements were placed at the extremes and others near the middle. This talking time notionalized the sort and added depth to the quantitative data. The entire process thus yielded a detailed personal configuration of viewpoints, where each of the 32 statements received a unique score between +5 and −5, effectively mapping the participant's subjective landscape onto an empirically analyzable structure.

In the fourth stage, the PQ method (2002) was employed to analyze the collected 53 Q sorting samples. This study used Principal Component Analysis to extract factors to obtain each sample's eigenvalues and explanatory ratios. We selected the first four factors for subsequent factor rotation and factor analysis, which met the standard conditions proposed by Watts and Stenner, that is, if the eigenvalue is greater than 1, the cumulative explained sample proportion is 54.74%, which meets the basic requirements of Q-analysis. This result indicates that the first four factors are significant for the factor analysis results (Watts and Stenner, 2012).

Next, the Varimax Rotation of the Factors method is chosen for factor rotation. The varimax rotation aligns with the core objective of Q methodology: to reveal the distinct viewpoints within a participant group. It produces a mathematically optimal solution by maximizing the variance explained by the extracted factors, resulting in clearer and more interpretable factor structures. Moreover, varimax relies directly on the topography of the correlation matrix, ensuring that the emergent factors are grounded in participants' own responses. This prioritization of participant input resonates with the qualitative ethos underlying much of Q methodological research (Watts and Stenner, 2012).

Then, the explanatory ratio of each factor to the total variable and the respondents' loading by factor can be obtained. According to the standard of the Q method, if the respondents' loading by factor is greater than 2.5$/\sqrt{Q\;sample\;size\;}$ (32), then this respondent is included in the factor. Otherwise, it is not included in the factor (Watts and Stenner, 2012). Therefore, the respondents' loading by factor in this study can only be attributed to this factor if they are more significant than 0.456. A single factor with both positive and negative significant loadings is termed ‘bipolar'. Crucially, these negative loadings must be retained as they represent a diametrically opposed viewpoint. The statistical significance is determined by the absolute value of the loading, but the sign reveals the direction of agreement. Thus, a single bipolar factor requires two distinct interpretations: one for the configuration defined by the positive loadings, and another for its mirror-image defined by the negative loadings (Watts and Stenner, 2012). If the respondent can be classified into two or more factors simultaneously, the respondent needs to be deleted. Finally, the factor is retained when the effective respondents within the factor are ≥2 (Watts and Stenner, 2012). After deleting respondents who were not loaded or invalid (marked with shadows in the figure), all four factors were ultimately retained and cumulatively explained 81% of the total variance (exceeding the standard of 34%−40%), which was considered sufficient (Watts and Stenner, 2012).

## 4 Results

From the results of factor analysis, there are four patterns or tendencies in college students' motivation to engage in online hot topics. Since researchers typically name factors based on discriminative statements and label each factor differently (Simons Joan, 2013), these four factors are ultimately named: self-referential rationalists, values-driven advocates, interest-oriented participants, and rights-protection oriented participants (see Table 1). Table 2 provides detailed statistical information about the four factors. Table 3 shows the Z-score and sorting of 32 statements on four factors. The interpretation is mainly based on the content of extreme values, while others serve as auxiliary explanations.

**Table 1:** Factor loadings for 53 Q sorts.

**Q SORT**	**Factor 1**	**Factor 2**	**Factor 3**	**Factor 4**
P2	−0.6153^*^	−0.0434	−0.1507	−0.1835
P5	0.2744	0.4129	0.3286	0.5684^*^
P6	0.7329^*^	0.0858	0.1856	0.4166
P7	−0.1633	0.0417	0.7181^*^	0.1332
P8	0.0423	0.5626^*^	0.3064	0.0032
P9	0.2163	0.1829	0.6244^*^	−0.0622
P10	−0.0124	−0.2310	0.7728^*^	0.2736
P12	0.4679	0.1851	0.4856^*^	−0.0300
P13	0.4073	0.3875	0.5315^*^	0.3003
P15	0.5578^*^	0.0841	0.1087	0.2066
P16	0.1244	0.0810	0.5512^*^	0.2049
P17	0.3552	0.1785	0.5046^*^	0.0353
P18	0.3586	0.0128	0.8069^*^	−0.2199
P19	0.4024	−0.0463	0.5543^*^	0.1085
P21	0.3698	0.7021^*^	−0.1476	−0.0312
P22	0.7477^*^	0.0137	0.0879	−0.0104
P23	0.0153	0.1198	0.6638^*^	0.3808
P24	0.4060	0.6729^*^	−0.2402	0.1264
P26	0.4945^*^	0.2713	0.0367	0.1619
P27	0.1597	0.0726	0.1389	0.6580^*^
P28	0.2076	0.3814	−0.0190	0.5465^*^
P30	0.2954	0.2441	0.6299^*^	0.2420
P31	−0.1319	0.3553	0.7083^*^	−0.0677
P32	−0.2864	−0.2396	−0.2751	0.4829^*^
P33	−0.0422	0.0935	0.7847^*^	0.2557
P34	0.1427	0.6729^*^	0.0187	0.2954
P35	0.3997	−0.1042	0.5587^*^	0.3458
P36	0.4363	0.0472	0.2605	0.6281^*^
P37	0.6300^*^	0.1388	0.3936	−0.1870
P38	0.2100	0.2251	0.4303	0.6790^*^
P40	0.8300^*^	0.0596	0.1291	0.0805
P41	0.4082	0.2648	0.6935^*^	0.1430
P42	0.0054	0.7528^*^	0.3103	0.0782
P43	−0.0734	0.7844^*^	0.3440	−0.0565
P44	0.0516	0.7005^*^	−0.2128	−0.0391
P45	−0.2826	0.5387^*^	0.3523	0.0357
P46	0.0531	0.5678^*^	0.0192	0.1834
P47	0.3418	0.5875^*^	−0.0519	0.1938
P48	−0.1869	−0.1706	0.6494^*^	−0.1538
P50	0.2698	0.6078^*^	0.3971	0.0394
P51	0.0503	0.2752	0.5421^*^	0.2396
P52	0.2688	0.0014	0.6197^*^	−0.1317
P53	0.2528	−0.0464	0.4990^*^	0.3097
P1	0.4149	0.1694	−0.0446	0.1605
P3	0.1897	0.4122	0.3989	0.1002
P4	0.4164	0.4542	−0.1045	0.4120
P11	−0.5068	0.0265	0.5165	0.2830
P14	0.2528	−0.0060	0.2391	0.3380
P20	−0.2219	0.4556	0.1386	0.4568
P25	0.0438	0.4731	0.0720	0.5064
P29	0.5962	0.6249	0.3100	0.2548
P39	−0.2958	0.3536	0.3806	−0.2512
P49	0.3401	0.3498	0.0979	0.4557

If the load factor of the responder is greater than 0.456, it will be manually marked as ^*^.

**Table 2:** The statistical details of four factors.

**Metric**	**Factor 1**	**Factor 2**	**Factor 3**	**Factor 4**
No. of defining variables	7	11	19	6
Average rel. coef.	0.800	0.800	0.800	0.800
Composite reliability	0.966	0.978	0.987	0.960
S.E. of factor Z-scores	0.186	0.149	0.114	0.200
% expl. var.	13	14	19	9

**Table 3:** Z-scores of 32 statements with corresponding ranks.

**No**.	**Statement**	**Factor 1**		**Factor 2**		**Factor 3**		**Factor 4**	
		**Z-score**	**Rank**	**Z-score**	**Rank**	**Z-score**	**Rank**	**Z-score**	**Rank**
1.	I am very confident in my answer to this topic.	1.291	3	−0.548	22	−0.429	23	0.167	17
2	I want to influence the development process of the event by engaging in the topic.	0.043	16	0.297	12	−1.633	30	0.441	14
3	I want to put pressure on relevant institutions through my own engagement.	0.253	15	−0.622	23	−2.167	32	0.971	5
4.	To keep this topic hot, i need to help.	0.987	7	1.084	6	−0.869	26	1.400	2
5.	I am the protagonist of society, so i want to engage in online hot topics.	−0.789	25	0.108	16	−1.823	31	−1.196	28
6.	I want to speak up for the weak and speak up for justice.	0.368	14	1.553	3	0.200	14	0.916	8
7.	I have experienced similar things and feel it.	1.883	1	−0.660	24	1.156	5	1.211	3
8.	It concerns my benefits, so i need to engage in.	0.983	8	−0.845	26	0.909	7	1.462	1
9.	I am worried that the same event may happen in the future.	0.803	9	0.752	8	1.184	4	0.918	7
10.	I got involved in it just to find out the truth.	−0.376	19	0.362	11	0.860	9	0.891	9
11.	I usually engage in discussions as a bystander.	−1.254	29	0.103	17	0.665	10	1.027	4
12.	I want to express my emotions and attitude toward the topic.	0.524	12	0.787	7	0.282	13	0.931	6
13.	Engaging in behavior can prove my sense of social responsibility.	−0.711	23	1.184	5	−0.520	24	−0.237	20
14.	I want to showcase an ideal image to others by engaging in hot topics.	−0.954	26	−1.409	30	−1.470	29	−1.488	31
15.	I want to get posts and likes through topic engagement.	−0.627	21	−1.919	32	−1.109	28	−2.156	32
16.	I want to engage in and pass the time.	−1.561	31	−0.686	25	0.056	17	−1.169	27
17.	The discussion was very intense, and i also want to engage in.	−0.543	20	−0.246	19	0.053	18	−1.061	26
18.	I strongly agree with a blogger or viewpoint, so i joined in.	0.714	11	−1.195	28	1.188	3	0.718	11
19.	Usually, the majority of people's views are mine.	−1.579	32	−1.063	27	−0.331	20	0.306	16
20.	Everyone around me has engaged in, and i need to stay consistent with them.	−1.402	30	−1.519	31	−0.872	27	−1.052	25
21.	I don't agree with the views of others, so i need to correct them.	1.232	5	−1.327	29	−0.144	19	−0.868	24
22.	It is very convenient to engage in discussions using mobile devices.	−0.301	18	0.721	9	0.869	8	0.750	10
23.	We media encourages young people to actively engage in public activities.	−0.648	22	0.269	13	0.152	15	−0.167	19
24.	The anonymity of the internet has increased my courage and enthusiasm.	−0.081	17	−0.372	20	−0.365	22	−0.752	23
25.	I often make comments on this app.	−1.222	28	−0.494	21	−0.689	25	−1.329	29
26.	The continuous push of apps has forced me to pay attention to and engage in it.	−1.173	27	0.389	10	0.095	16	0.592	13
27.	The ranking of hot searches determines which topics i engage in.	−0.757	24	0.187	15	−0.353	21	−1.449	30
28.	This topic is of interest to me.	1.370	2	1.240	4	1.898	1	0.699	12
29.	I find a comment under this topic very interesting.	0.410	13	0.220	14	1.404	2	0.424	15
30.	I engaged in because i have a knowledge background in the topic content.	1.044	6	−0.128	18	1.080	6	−0.259	21
31.	My values require me to actively engage in when encountering similar topics.	1.280	4	2.009	1	0.283	12	−0.674	22
32.	I want to express my values.	0.792	10	1.767	2	0.440	11	0.033	18

Colors indicate the ranking of the statement's score in the factor, highlighting the top three and bottom three, to help identify the factor.

### 4.1 Factor 1: self-referential rationalists

Factor 1 accounted for 13% of the study variance and was defined by the 7 respondents loading on it as “self-referential rationalists.” The configuration of this factor suggests that while the positively scored statements encompass multiple dimensions—including self-relevance, self-efficacy, personal interests, knowledge, and values—they are unified by a central focus on the self. Conversely, statements related to the mediating role of social support were unanimously assigned extreme negative scores. This pattern indicates that the engagement motivation for self-referential rationalists stems primarily from internal drives, rather than external social influences. This finding resonates with social identity theory, which posits that group memberships shape self-concept and can influence behavior (Abrams and Hogg, 2010); here, however, the individuals' behavior appears to be guided more by personal identity factors than by salient group identities. These participants emphasized the importance of a topic's connection to their personal experiences, benefits, or future concerns (S7, +5; S8, +2; S9, +2). Their level of interest in the topic, possession of relevant knowledge (S28, +4; S30, +3), and confidence in their own viewpoints (S1, +4) were also critical factors prompting their engagement on social media. A distinctive characteristic of this group is their pronounced rationality and resistance to external pressure. They reported that they would not participate simply to conform with the majority or their immediate social circle (S19, −5; S20, −4), nor would they engage casually to pass the time or due to algorithmic content (S16, −4; S26, −3). This sentiment was encapsulated by Respondents 15, 26, and 40, who unanimously stated: “We should not be constrained by the intelligence of the mobile Internet or the actions of others. I usually browse much information to obtain knowledge. Unless the topic has much to do with me or I am very interested, I will give my opinion instead of following the crowd.”

### 4.2 Factor 2: values-driven advocates

Factor 2 was defined by 11 respondents, accounting for 14% of the study variance, and was labeled “Values-driven advocates.” The factor array highlights the dominant role of values, particularly justice, in motivating engagement with social media hot topics. This motivational profile is strongly aligned with the value-belief-norm theory (Zhang et al., 2025), which suggests that strongly held values (like justice) can activate personal norms and a sense of moral obligation, thereby motivating pro-social actions.

Data from the Q sorts and subsequent interviews reveal that these value practitioners possess a strong sense of justice and are actively committed to pursuing social fairness (S6, +4). Their engagement is primarily driven by this value (S31, +5), with the aim of protecting the vulnerable and upholding social justice through their own words and actions, thereby expressing and practicing their core beliefs (S32, +4). This process also serves as a way for them to demonstrate a sense of social responsibility (S13, +3). As Respondents 24 and 45 articulated: “Whenever I see events like power bullying the weak on social media, I always feel sad and angry. The strong sense of justice in my heart drives me to help the vulnerable through my actions so that those who make mistakes receive the punishment they deserve. This process makes me feel my value and strength because I have fought for the rights and justice of others.”

Driven by their strong moral stance, these participants expressed strong opposition to individuals who engage with online hot topics for personal gain or attention, consistently assigning negative scores to statements reflecting utilitarian motives (e.g., S15, −5; S14, −4; S09, −4; S22, −3). This disapproval was a recurring theme in the interviews. Respondent 21 stated, “Many people use online hotspots to gain attention, and their values are problematic. I am not one of them.” Similarly, Respondent 24 emphasized, “I can't stand it when people fish for clout in serious discussions—it disrespects the issue and the people affected,” and Respondent 8 added, “Engaging should be about truth and fairness, not personal advantage.” These comments collectively underscore that ethical principles, rather than instrumental benefits, are the core motivators for this group.

### 4.3 Factor 3: interest-oriented participants

Factor 3 was defined by 19 respondents, accounting for 19% of the study variance, and was labeled “interest-oriented participants.” The factor array indicates that for this group, the primary driver of engagement is interest fit, while perceptions of self-efficacy are notably low. This pattern of media selection based on fulfilling specific gratifications aligns with the Use and Gratification (U&G) model, which posits that users actively choose media to satisfy psychological needs (Wei et al., 2024).

The central motivation for interest-oriented participants is their personal interest in a topic. This encompasses interest in the topic itself (S28, +5), the associated commentary (S29, +4), and the views of certain opinion leaders (S18, +4). This sentiment was echoed by Respondents 30, 31, and 35, who unanimously agreed, “I will browse much information, but I will only engage in interesting topics.” Respondent 9 added, “The purpose of going online is entertainment, and whether or not to participate naturally depends on whether it attracts my attention and stimulates my interest.”

A distinctive aspect of this factor is the participants' markedly low sense of self-efficacy. They perceive their ability to influence societal issues as limited, viewing themselves as “ordinary college students” with minimal impact. They believe their strengths are too weak to allow them to become prominent actors in social discourse (S5, −4), exert pressure on others (S3, −5), or affect the development of public events (S2, −4). As Respondent 18 poignantly stated, “Many people online advocate for others' grievances. However, I feel like a feather, and words have no power, making it difficult to influence others' actions.” This perceived lack of agency contrasts with their active selection of content based on interest, highlighting a consumption-oriented rather than change-oriented engagement style.

### 4.4 Factor 4: rights-protection oriented participants

Factor 4 was defined by 6 respondents, accounting for 9% of the study variance, and was labeled “rights-protection oriented participants.” A defining characteristic of this group is a strong sense of self-efficacy, contrasting sharply with the previous factor. They are confident that their engagement can effectively safeguard their own interests or those of others, which constitutes their primary motivation.

The Q-sorts from this group show high scores on statements related to self-interest, self-efficacy, and proactive engagement. They are highly concerned with whether a topic impacts their personal interests (S8, +5) and participate to express personal experiences and future concerns (S7, +4; S9, +2). As Respondent 28 stated, “I usually do not comment, but if the topic is related to my benefit. I want to attract more attention through participation so that the development of the situation is more beneficial to myself.”

Beyond self-interest, their motivation extends to collective action. They engage to maintain a topic's popularity (S4, +4), attract broader attention to exert pressure on relevant authorities (S3, +3), and ultimately help vulnerable groups protect their rights (S6, +2). Respondent 7′s comment illustrates this strategy: “My ‘um' is very much needed… Millions of ‘helpers' will make certain departments pay attention to this matter.”

This behavior can be further understood through the corrective action hypothesis (Rojas, 2010). When individuals perceive the prevailing opinion on a topic to be incorrect or unjust, they may speak out to “correct” the public discourse. For rights-protection oriented participants, their assertive engagement serves not only to defend interests but also to counter what they see as inaccurate or unfair narratives, aligning with observations that people express opinions to correct others' views (Megan et al., 2020). Thus, their participation is driven by a combination of perceived efficacy and a corrective impulse within the public sphere.

## 5 Discussion

Table 4 shows the relationship between the results of four factors in the results and the six-dimensional motivation extracted in the literature review. Factor 1 strongly correlates with self-efficacy, the correlation of the topic to “me”, and knowledge. Factor 2 highly emphasizes values—and factor 3 favors interest. Factor 4 is similar to factor 1 in emphasizing common aspects: self-efficacy and the topic's relevance to “me”, but factor 1 emphasizes the “self” more while factor 4 focuses more on benefit. This reflects that the internal combination of specific engagement motivations is specific, differentiated, and distinctive. Therefore, it triggers thinking about the internal generation logic of these four different engagement motivations.

**Table 4:** The relationships between four factors and six dimensions identified in the literature.

**Naming**	**F1: self-referential rationalists**	**F2: values-driven advocates**	**F3: interest-oriented participants**	**F4: rights-protection oriented participants**
1. Self-efficacy	+	–	–	+
2. The relevance of the topic to “me”	+	/	/	+
3. Psychological empowerment	/	–	/	–
4. The mediating effect of social support	–	/	/	/
5. The impact of new media technology	/	/	/	/
6. Interest, knowledge, and value	knowledge +	Value +	Interest +	/

The notion “+” “/” “-” represent three different meanings: strong relations, weak relations and with no relations.

The self-referential rationalists identified in this study exemplify a rational and self-directed approach to online engagement among university students. This pattern of behavior can be contextualized within a broader digital environment that is often characterized by diverse, entertainment-oriented content and a plurality of values. In such a context, the observed factor suggests that some students adopt a highly independent stance. Their engagement decisions are predominantly influenced by internal benchmarks—such as personal experiences, interests, confidence, and values—rather than by external social pressures or prevailing opinions. Notably, personal experience emerged as a particularly critical factor in their Q-sorts and interview responses. These participants often articulate their views by sharing personal narratives and reflections, a practice that may serve to enhance the perceived credibility and persuasiveness of their contributions, even when their primary motivation is self-expression.

Values-driven advocates are characterized by a strong emphasis on justice. This orientation can be understood within a broader societal context where the pursuit of fairness and justice is increasingly prominent. For these individuals, often influenced by value-based education, social justice becomes a core ideal. Their engagement is typically triggered by exposure to incidents of perceived injustice on social media, which creates a sense of moral obligation to speak up for the vulnerable. While this study's data, including participant interviews, confirm that such value-driven participation aims to influence real-world outcomes and draw attention to issues of fairness, it is also important to note its potential dual nature. As observed in broader online phenomena, this type of involvement, despite its positive intentions, can sometimes adopt a confrontational tone and, in certain cases, contribute to polarized public debates.

Interest-oriented participants represent a common archetype in this study, underscoring the fundamental role of personal interest as a motivator for online engagement among university students. These participants actively select topics and commentary that align with their personal interests, engaging through responses, expressions of agreement, or debate. In the vast and diverse digital landscape, they are drawn to participate when topics resonate with their passions. A key finding from this study is that even when these individuals perceive their self-efficacy as low and doubt their capacity to influence broader outcomes, their intrinsic interest alone provides sufficient motivation for participation. Consequently, while not explicitly aiming to shape public opinion, the collective activity of such participants constitutes a significant form of online engagement that contributes to the vitality and direction of digital discourse.

Rights-protection oriented participants are characterized by a high sensitivity to issues of benefit and rights, demonstrating a willingness to defend both personal and collective interests. While sharing a strong sense of self-awareness with self-referential rationalists, their focus extends outward to safeguarding collective rights and assisting others. Equipped with strong self-efficacy, they engage with confidence and agency. This pattern of behavior can be contextualized within a broader societal phase of deepened reform, where social restructuring involves complex realignments of interests. In such a context, online platforms often become arenas for voicing concerns, particularly when offline channels are perceived as inadequate. It is in this environment that rights-protection oriented participants emerge to support and amplify marginalized voices.

Furthermore, this study observes that these participants often embody the rational and responsible online engagement encouraged in contemporary China. Their actions reflect how bottom-up feedback can contribute to public discourse. However, while affirming the value of such participation, it is also pertinent to note that constructive expression is most effective within reasonable boundaries. Therefore, fostering a healthy digital public space may involve guidance that balances autonomy with social responsibility.

## 6 Conclusion

This study explores the differentiated motivations and behavioral patterns among college students when engaging with online trending topics, employing Q methodology to identify four distinct engagement profiles. The research was conducted with a group of students from 11 prefecture-level cities in Jiangsu Province. The first type, *self-referential rationalists*, are characterized by a strong self-orientation; they evaluate topics based on personal relevance, interest, and expressive accuracy, and are less susceptible to external influence. The second type, *values-driven advocates*, are motivated primarily by a sense of justice and moral principles. The third type, *interest-oriented participants*, selectively engage with content aligned with their personal interests. The fourth type, *rights-protection oriented participants*, are driven by the goal of safeguarding their own rights and those of others. Among these, self-referential rationalists and interest-oriented participants constitute a large portion of the sample and generally engage in a relatively calm manner. As Respondents 7 and 26 noted, “I just want to broaden my knowledge, find leisure and entertainment, and enjoy life through engaging with topics.” In contrast, values-driven advocates and rights-protection oriented participants tend to engage more assertively and purposefully.

The findings of this study provide valuable insights for digital citizenship education, student support services, and media literacy initiatives. Moving beyond one-size-fits-all approaches, educators and platform designers can develop differentiated strategies that account for these distinct motivational profiles. For instance, educational interventions could be tailored to enhance critical reflection on personal and collective values or to facilitate more constructive forms of advocacy concerning rights and justice. Ultimately, supporting students in developing the capacity to navigate complex online environments in a self-directed yet socially conscious manner can foster healthier and more inclusive digital discourse. The descriptive typology established here offers a foundational framework for future empirical research into the dynamics of youth online participation and the design of evidence-based educational interventions.

As far as we know, this study is the first to use the Q method to study the motivation of college students to engage in online hot topics in China. However, the Q method has limitations as it requires a relatively small sample size. The sample is restricted to one province with snowball recruitment; remote administration may affect sort behavior; the concourse may not cover platform-specific affordances (e.g., algorithmic curation, duets/stitching, livestream gifting). Propose follow-ups that test stability of viewpoints across regions, platforms, and time.

## Data Availability

The original contributions presented in the study are included in the article/supplementary material, further inquiries can be directed to the corresponding author.
